# Functional status of pediatric patients after discharge from intensive care units in a middle‐income country

**DOI:** 10.1111/ped.70300

**Published:** 2025-12-27

**Authors:** Regina Melittio Gasparetti, Kelsy Catharina Nema Areco, Heitor Pons Leite, Paulo Cesar Koch Nogueira, Tulio Konstantyner

**Affiliations:** ^1^ Universidade Federal de São Paulo/Escola Paulista de Medicina (EPM/UNIFESP) São Paulo Brazil

**Keywords:** chronic disease, critical care outcomes, functional status, pediatric intensive care unit

## Abstract

**Background:**

Patients discharged from the intensive care unit (ICU) often experience high morbidity rates. The aim of the present study was to estimate the incidence of functional capacity impairment in pediatric patients who were discharged from the ICU in a middle‐income country and to identify associated factors, considering the socioeconomic context.

**Methods:**

This was a multicenter cohort study of 357 patients aged <18 years admitted to three ICUs in the interior cities of São Paulo State. The Functional Status Scale was used to assess the patients at admission, ICU discharge, and hospital discharge. New morbidity was defined as a change in any domain of the scale equal to or greater than two, or a change in the total score equal to or greater than three. A multiple logistic regression model was used to identify independent associations with new morbidity.

**Results:**

New morbidity occurred in 14.6% and 12.3% of patients at ICU and hospital discharge, respectively, compared with those at admission. The feeding (*p* < 0.001) and respiratory (*p* = 0.036) functional domains were the most affected at ICU discharge. The risk factors associated with new morbidity at hospital discharge were heart disease, older age, longer ICU stay, and higher PELOD2 severity score.

**Conclusions:**

To reduce the risk of new functional morbidity, healthcare teams should be attentive to the critically ill pediatric population, particularly those who are older and have chronic clinical conditions, especially heart disease.

## INTRODUCTION

Biotechnological advancements, the creation of intensive care protocols, and humanization efforts have contributed to the development of Intensive Care Units (ICUs) with reduced mortality rates in recent decades. However, patients discharged from ICUs often experience high rates of morbidity according to functional status scales.^1^, ^2^

The incidence of functional impairment in the pediatric age group after ICU discharge varies widely, ranging from 4.8% to 82%, depending on the hospital care infrastructure, the patient's functional status at admission, clinical characteristics and disease severity.^3^, ^4^, ^5^, ^6^, ^7^, ^8^, ^9^, ^10^, ^11^, ^12^, ^13^, ^14^

Despite some studies focusing on the importance of low socioeconomic status in determining neurological and cognitive decline in critically ill pediatric patients, few studies have tested its association with functional loss after hospital discharge.^15^ Studies that mention or assess the influence of these conditions on the recovery of these patients have been conducted in countries with different cultural and socioeconomic characteristics than Brazil, which limits the generalizability of the findings.^16^, ^17^

In addition to household income, which is essential for managing patients' needs, the region or country of residence potentially affects functional loss determination. The conditions of this multifactorial outcome, which involves different hierarchical levels of healthcare, may not be the same in countries with other socioeconomic levels. In Brazil, studies have evaluated functional loss in pediatric patients. However, none of these studies tested the association with socioeconomic variables.^6^, ^7^, ^11^, ^12^, ^13^, ^14^

Therefore, the changing epidemiological scenario in pediatric ICUs with higher survival rates but frequent functional loss, and the need to deepen knowledge in developing countries like Brazil emphasizes the importance of studying not only the clinical characteristics of pediatric patients discharged from the ICU but also the socioeconomic and distal variables associated with functional loss after hospital discharge. This is particularly important for patients in the early stages of life, who usually require more attention from their families and society.^7^

Identifying specific socioeconomic factors can assist in the management of health services in areas with fewer resources, with the goal of improving patient and family care and quality of life. Establishing strategies to control these factors can prevent or reduce functional loss in pediatric patients during hospitalization.^11^, ^13^, ^14^, ^18^

This study aimed to estimate the incidence of functional capacity impairment in pediatric patients who have been discharged from the ICU from a middle‐income country and to identify associated factors, considering the socioeconomic context.

## METHODS

A prospective multicenter cohort study was conducted in three pediatric ICUs of referral hospitals in the interior cities of the Paraíba Valley, in the State of São Paulo: Dr. José de Carvalho Florence Municipal Hospital (10 beds), Fabiana de Macedo Moraes Treatment Center, Support Group for Children with Cancer‐CTFM‐GACC (5 beds), both in São José dos Campos, and São Francisco de Assis Hospital in Jacareí (10 beds). The municipal human development index (IDHM) of the municipalities in the Paraíba Valley varies from 0.655 (Natividade da Serra, ranked 3015th nationally) to 0.807 (São José dos Campos, ranked 25th nationally). Data were collected from March 12, 2019 to March 11, 2020, starting from admission to the Pediatric ICU until hospital discharge or patient death (follow‐up time).

The study sample consisted of patients aged <18 years admitted to the participating hospital ICUs. Patients with a corrected gestational age of less than 38 weeks, those with readmissions, and those whose parents/guardians did not consent to participate in the study were excluded. The number of patients recruited during the study period (exposed and non‐exposed =1:1) was considered sufficient to estimate risk ratios of approximately 1.5 (alpha = 0.05, beta = 0.20), with an estimated 45% prevalence of functional loss in the exposed group. On ICU admission, the parents/guardians were interviewed, and a structured questionnaire with socioeconomic, cultural, demographic and clinical information about the patients, such as pre‐existing chronic illnesses, was applied. Family income was estimated for economic classification using a questionnaire from the Brazilian Association of Research Companies (ABEP)^19^ and the IDHM of the patient's city of residence was used to reflect the socioeconomic environment. During follow‐up, data on hospitalization and clinical progression were collected, such as organ dysfunction assessed using The Pediatric Logistic Organ Dysfunction Score (PELOD2),^20^ recorded at ICU admission, which ranges from zero to 71 and indicates severity. The probability (%) of death was also estimated using the Pediatric Index of Mortality (PIM2)^21^ within the first 6 h of hospitalization. Additionally, the Glasgow Coma Scale (GCS), ranging from three to 15, resulting in lower values with a decreased level of consciousness, was used at admission.

Functional status was assessed using the Functional Status Scale (FSS), which was constructed and validated by Pollack et al. and translated and adapted to the Brazilian pediatric version by Bastos et al.^2^, ^6^ The FSS is based on daily living activities, is easy to apply, and is composed of elementary questions distributed across six domains: mental, communication, sensory, motor, respiratory, and eating. The score varies from 6 in patients with normal functional status to 30 in patients with severe dysfunction (vegetative state). Each domain received a score from one to five, with a value of one for adequate functional status and five for severe dysfunction. The FSS was applied in three time points by a single researcher upon admission and discharge from the ICU and hospital. Patients were classified by functional status as adequate when the FSS was ≤7, mild functional loss when the FSS was between 8 and 9, moderate functional loss when the FSS was between 10 and 15, severe functional loss when the FSS was between 16 and 21, and very severe functional loss when the FSS was >21. In the event of death, the highest severity score was assigned 30 according to FSS criteria.^5^, ^6^, ^9^

Changes in functional capacity were defined as the presence of new morbidities. New morbidity was defined as a change in a domain equal to or greater than two or a change in the total score equal to or greater than three between admission and discharge from the ICU and hospital. To study the association of independent variables with changes in functional capacity, the presence of new morbidity at hospital discharge was considered an outcome.^3^, ^4^ Categorical variables were described as absolute and relative frequencies and continuous variables as median (Md) and interquartile range (IQR). The Kolmogorov–Smirnov test was used to evaluate the normality of the distribution of the numerical variables. Friedman's analysis of variance was used to compare the median FSS scores at the three time points. To define the differences between time points, post‐hoc analysis was performed using the Wilcoxon test (signed‐rank) and Bonferroni correction.

To study the association between the explanatory variables and the outcome (new morbidity at hospital discharge), the chi‐square and Fisher's exact tests were used for categorical variables, and the Student's *t*‐test and Mann–Whitney tests were used to compare means and medians, respectively, and according to the normality characteristics of the tested variables. Additionally, a multiple logistic regression model was used. The explanatory variables that presented *p* values < 0.20 in the univariate analysis were chosen for inclusion in the multiple model. The variable inclusion method was stepwise backward (Wald test). A level of 0.05 (*α* error 5%) was chosen to indicate a statistically significant association, being the criterion used to remain in the final model. Furthermore, to avoid multicollinearity between the independent variables, only those that did not show a linear relationship were maintained.

The Statistical Package for the Social Sciences for Windows was used for all statistical analyses. The study was approved by the Ethics and Research Committees of the three hospitals and Federal University of São Paulo. Parents/guardians read and signed the Free and Informed Consent Form, and participants read (or were read) and signed the Free and Informed Consent Form, when relevant, in accordance with Resolution 466/2012 and Operational Standard No. 001/2013 of the National Health Council.

## RESULTS

A total of 357 patients were included in this study. The majority were male (57.4%), 9 (2.1%) were newborns, 147 (38.3%) were infants, 99 (28.8%) were preschoolers, 63 (19%) were schoolchildren, and 39 (11.7%) were teenagers. Hospitalizations occurred through the Unified Health System (SUS) in 234 patients (65.5%). With regard to socioeconomic status, 257 patients (73%) belonged to the lower classes (C1, C2, D, or E), and 249 (70.7%) of the household heads had greater than or equal to secondary education graduation (Table 1).

**TABLE 1:** Prevalence of characteristics of patients admitted to the Intensive Care Unit of three hospitals in the State of São Paulo.

Variable		*n*	% (95% CI)
Sex	Male	205	57.4 (52.1–62.6)
	Female	152	42.6 (37.4–47.9)
	Total	357	100
Gestational age (weeks)	<37	62	19.1 (15.0–23.8)
	≥37	262	80.9 (76.2–85.0)
	Total	324	100
ABEP	A, B1, B2	95	27.0 (22.4–31.9)
	C1, C2, DE	257	73.0 (68.1–77.6)
	Total	352	100
Education of the family household heads	Incomplete secondary education	103	29.3 (24.6–34.3)
	Completed high school at least	249	70.7 (65.7–75.4)
	Total	352	100
Source of payment	SUS	234	65.5 (60.4–70.5)
	Health insurance	123	34.5 (29.5–39.6)
	Total	357	100
Hospitalization	Clinic	258	72.3 (67.3–76.9)
	Surgical	99	27.7 (23.1–32.7)
	Total	357	100
Surgical ward	No	281	78.7 (74.1–82.8)
	Yes	76	21.3 (17.2–25.9)
	Total	357	100
Cancer patient	No	303	84.8 (80.7–88.4)
	Yes	54	15.2 (11.6–19.3)
	Total	357	100
Pre‐existing chronic diseases	No	108	30.3 (25.5–35.3)
	Yes	249	69.7 (64.7–74.5)
	Total	357	100
Type of pre‐existing chronic diseases^a^	Cardiac	35	9.8 (6.9–13.4)
	Total	357	100
	Neurological	111	31.1 (26.3–36.2)
	Total	357	100
	Kidney	15	4.2 (2.4–6.8)
	Total	357	100
	Respiratory	72	20.2 (16.1–24.7)
	Total	357	100
	Others	247	69.2 (64.1–73.9)
	Total	357	100

Abbreviations: ABEP, socioeconomic classification of the Brazilian Association of Research Companies; CI, confidence interval; SUS, Unified Health System.

^a^ The number and percentage of each pre‐existing chronic diseases in relation to the total sample size (*n* = 357).

The incidence of death during hospital admission in the studied sample was 7.6% (*n* = 27). The median age of patients at admission was 2.83 years (IQR = 7.05), median length of ICU stay was 6 days (IQR = 9), MHDI of the patients' city of residence was 0.807 (IQR = 0.03), risk of death (PIM2) was 1.2% (IQR = 3.4), and PELOD2 and Glasgow scores were 1 (IQR = 10) and 15 (IQR = 5), respectively.

The FSS was applied to all 357 patients at three time points (ICU admission, discharge, and hospital discharge/death). Upon admission to the ICU, 23.5% (*n* = 84) of the patients already had some functional impairment, 5.3% had mild functional losses, and 18.2% had moderate to very severe functional losses. At discharge from the ICU and at hospital discharge, 30.5% (*n* = 109) and 34.2% (*n* = 122) of patients had some functional loss.

**FIGURE 1:**
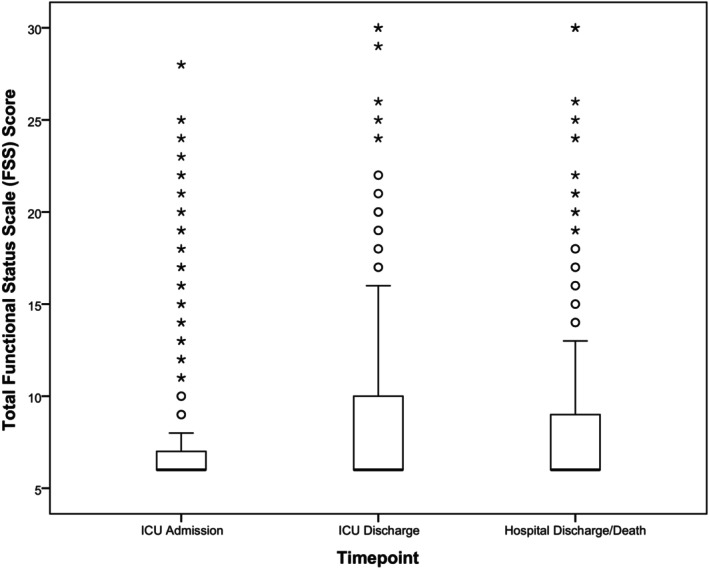
Box plot of the variation in the Functional Status Scale (FSS) at the three time points (admission to the Intensive Care Unit, discharge from the Intensive Care Unit, and hospital discharge/death) of patients admitted to the pediatric Intensive Care Unit of three hospitals in the State of São Paulo. Friedman analysis of variance (*p* < 0.001).

Figure 1 shows the variation in the median of the total FSS scores at the three time points of the study (*p* < 0.001). The complementary multiple comparison test showed the greatest differences between the time of admission (Md: 6.0, IQR: 6.0–7.0) and discharge from the ICU (Md: 6.0, IQR: 6.0–10.0) and from admission to hospital discharge (Md: 6.0, IQR: 6.0–9.5) (*p* < 0.001). The food (*p* < 0.001) and respiratory (*p* = 0.036) domains were the two most compromised domains at discharge from the ICU in relation to hospitalization.

The incidence rates of new morbidity at discharge from the ICU and hospital in relation to admission were 14.6% (*n* = 52) and 12.3% (*n* = 44), respectively. New morbidity at hospital discharge in relation to admission was less frequent in the “communication” domain (8.1% – 95% CI: 5.5–11.5) and more frequent in the “food” domain (9.8% – 95% CI: 6.9–13.4); however the variation between domains was small.

Of the patients who did not present with new morbidity at hospital discharge (*n* = 313), the majority (*n* = 303) did not develop new morbidity at discharge from the ICU. The other patients (*n* = 10) developed new temporary morbidity during hospitalization but recovered functional status between ICU discharge and hospital discharge. Furthermore, two patients who presented with new morbidity at hospital discharge (loss after discharge from the ICU) did not have it at discharge from the ICU in relation to admission.

The association between the variables studied and the new estimated morbidity at hospital discharge in relation to ICU admission is presented in Table 2. The type of care (*p* = 0.037), cancer (*p* = 0.001), and chronic heart disease (*p* = 0.046) were associated with new morbidity. Table 3 presents comparisons of medians between groups with and without new morbidities. Statistically significant differences were observed between median age (*p* < 0.011), ICU length of stay (*p* < 0.001), PIM2 (*p* = 0.001), PELOD2 (*p* = 0.001), and Glasgow scale (*p* = 0.011).

**TABLE 2:** Prevalence of factors associated with new morbidity at hospital discharge, in relation to admission to the Intensive Care Unit of pediatric patients from three hospitals in the State of São Paulo.

Factor		*n*	Functional loss				*ꭓ* ^2^; *p*‐value
			No		Yes		
			%	*n*	%	*n*	
Sex	Male	205	85.9	176	14.1	29	1.478; *p* = 0.224
	Female	152	90.1	137	9.9	15	
Prematurity	Yes	62	91.9	57	8.1	5	1.461; *p* = 0.227
	No	262	86.3	226	13.7	36	
ABEP	A, B1, B2	95	90.5	86	9.5	9	0.748; *p* = 0.387
	C1, C2, D e E	257	87.2	224	12.8	33	
Education of the family household heads^a^	No	103	83.5	86	16.5	17	2.898; *p* = 0.089
	Yes	249	90.0	224	10.0	25	
Source of payment	SUS	234	85.0	199	15.0	35	4.355; *p* = 0.037
	Health insurance	123	92.7	114	7.3	9	
Hospitalization	Clinic	258	86.4	223	13.6	35	1.326; *p* = 0.250
	Surgical	99	90.9	90	9.1	9	
Cancer patient	No	302	90.4	273	9.6	29	11.494; *p* = 0.001
	Yes	54	74.1	40	25.9	14	
Pre‐existing chronic diseases	No	108	90.7	98	9.3	10	1.347; *p* = 0.246
	Yes	249	86.3	215	13.7	34	
Cardiac	No	322	88.8	286	11.2	36	3.983; *p* = 0.046
	Yes	35	77.1	27	22.9	8	
Neurological	No	246	89.4	220	10.6	26	2.257; *p* = 0.133
	Yes	111	83.8	93	16.2	18	
Kidney	No	342	87.7	300	12.3	42	0.015; *p* = 0.903
	Yes	15	86.7	13	13.3	2	
Respiratory	No	285	87.4	249	12.6	36	0.123; *p* = 0.726
	Yes	72	88.9	64	11.1	8	

Abbreviations: ABEP, socioeconomic classification of the Brazilian Association of Research Companies; Prematurity: gestational age at birth less than 37 weeks; SUS, Unified Health System; *ꭓ* ^2^, Chi‐square test.

^a^ High school graduated.

**TABLE 3:** Median, interquartile range, maximum and minimum values of factors associated with new morbidity at hospital discharge, in relation to admission to the Intensive Care Unit of pediatric patients in three hospitals in the State of São Paulo.

	NM	*n*	Median	Minimum	Maximum	IQR	*U*; *p*‐value
Age (years)	No	313	2.35	0.03	1798	6.49	5246.0; *p* = 0.011
	Yes	44	5.98	0.08	17.90	10.76	
Length of ICU stay (days)	No	313	6	1	60	7	3770.0; *p* < 0.001
	Yes	44	14.5	1	71	24	
PIM2 (%)	No	313	1.1	0	55	2	3516.5; *p* < 0.001
	Yes	43	4.8	0	88	14	
PELOD2	No	313	1.00	0	33	10	3489.0; *p* < 0.001
	Yes	43	11.00	0	42	19	
Glasgow IDHM	No	313	15.00	3	15	5	5418.5; *p* = 0.011
	Yes	44	13.00	3	15	12	
	No	312	0.807	0.655	0.807	0.03	6231.0; *p* = 0.276
	Yes	44	0.800	0.655	0.807	0.03	

Abbreviations: Glasgow, Coma scale; ICU, Intensive Care Unit; IDHM, Municipal Human Development Index; IQR, interquartile range; NM, new morbidity (functional loss); PELOD2, The Pediatric Logistic Organ Dysfunction score; PIM2, Pediatric Index of Mortality; U, Mann–Whitney test.

In the multiple logistic model, the independent variables that remained in the final model, as they maintained a risk association (*p* < 0.05) with new morbidity at hospital discharge in relation to ICU admission, were diagnosis of heart disease, age in years, length of ICU stay, and PELOD2 severity score (Table 4).

**TABLE 4:** Multiple logistic model of factors associated with new morbidity at hospital discharge, in relation to admission to the Intensive Care Unit of patients from three hospitals in the State of São Paulo.

Variable		OR (95% CI)	*p*‐value
Heart disease	Yes	3.38 (1.11–10.27)	0.032
	No	1.00	
Age (years)		1.18 (1.09–1.28)	<0.001
Length of ICU stay (days)		1.08 (1.05–1.12)	<0.001
PELOD2		1.09 (1.05–1.14)	<0.001

Abbreviations: CI, confidence interval; ICU, Intensive Care Unit; OR, odds ratio; PELOD2, The Pediatric Logistic Organ Dysfunction score.

## DISCUSSION

The estimated incidence of new morbidity was 14.6% at ICU discharge and 12.3% at hospital discharge. The latter was similar in the six FSS domains, ranging from 8.1% (communication) to 9.8% (food).

Approximately two‐thirds of the services were delivered by the SUS, and two‐thirds of the patients' families belonged to the lowest socioeconomic strata (C1, C2, D, or E). The largest age group consisted of children under 2 years old, and most of these children were male (57.4%).

The variables associated with a higher risk of new morbidity at hospital discharge were older age, higher PELOD2 severity score, longer ICU stay, and the presence of heart disease.

Other factors examined, like income, education, and healthcare payment did not show association with new health morbidity in the final logistic regression model. This can be attributed to the homogeneous characteristics of the sample studied, as all patients lived in the Paraiba Valley, where the participating hospitals are located. However, in another study it was found that socioeconomic factors were associated with cognitive dysfunction after ICU discharge.^22^ This indicates that these characteristics may play a more significant role in a patient's health after hospitalization than during their hospital stay. Consequently, this period demands greater resources to ensure adequate outpatient follow‐up and access to rehabilitation therapy.

The median age in our study was higher than that reported by Pereira et al. (1.58 years) and Alievi et al. (1 year).^6^, ^11^ This difference may be related to the specificities of care services and the clinical characteristics of patients treated, which vary depending on the referral system, structure, and complexity of care in each ICU. For patients presenting with new morbidity, the median age was approximately 6 years. In a multicenter study of 5017 patients, Pollack et al. found a median age of 3.7 years, with new morbidity being more common in young infants.^4^ In contrast, Maddux et al. reported a median age of 7.1 years among children treated at a trauma hospital who developed new morbidity.^10^ The higher median age observed in our study compared to that of Pollack et al.^4^ is likely attributable to the varied characteristics of the population studied, which included trauma victims, individuals who attempted suicide, and those with oncological diseases, conditions more prevalent among schoolchildren and adolescents.

Upon admission to the ICU, approximately 70% of the patients had comorbidities, and about one‐quarter experienced some degree of functional loss. During the ICU stay, 14.6% of the patients developed new morbidities. In a study by Matics et al., it was found that 37% of patients had comorbidities, 44.8% exhibited some degree of dysfunction upon admission, and around 8.6% developed new morbidities by discharge at a university hospital in Chicago, USA.^15^ The differences between the studies likely stem from the specific socioeconomic and cultural characteristics of the populations, variations in clinical criteria for ICU admission, the availability of care resources, and, importantly, the rehabilitation support provided after discharge, which was offered by this American hospital.^15^

Other studies conducted in southern Brazil estimated new morbidity rates to be higher than those found in the present study. According to the FSS, 82% and 77.8% of patients were reported to experience new morbidity at hospital discharge.^6^, ^13^ Another study using POPC and PCPC scales identified cognitive impairment in 60% of children and functional damage in 85% at ICU discharge.^11^ These high incidences can be explained by the characteristics of the patients treated in ICUs at university and referral hospitals, which cater to critically ill patients with chronic diseases.

The present study found a new morbidity rate at hospital discharge that was nearly three times higher than the 4.8% reported by Pollack et al.^4^ This discrepancy might be linked to differences in the quality of care, infrastructure and resource availability at the hospitals where patients were recruited.

The mortality rate also varied greatly between studies. In our study it was 7.6%, whereas the rates found in other studies ranged from 2% to 12%.^4^, ^6^, ^11^, ^13^ Interestingly, one study found an incidence of morbidity twice as high as the mortality rate, which is a proportion similar to that found in our study.^4^ This emphasizes the importance of preventing functional loss, as this can compromise not only quality of life, but also patient survival.

The studies also differed in terms of which domains of the FSS scale were most affected. One study indicated that the “respiratory” and “nutrition” domains showed greater functional loss.^12^ In another study, the most affected domains were the “motor” and “food” domains.^6^ A third study showed that the most affected domains were similar to those in our study: feeding, motor, and respiratory.^13^ Nevertheless, the differences between the domains in the studies are minor and the greater predominance of one domain or another is probably linked to the clinical characteristics of the patients, thus not requiring the inclusion of domain‐specific health care strategies.

Regarding heart disease, specifically, Medeiros et al. found 42.8% of patients undergoing heart surgery with new morbidity upon discharge from the ICU.^12^ In our sample, the risk of new functional morbidity was 3.5 times higher in patients with heart disease. This can be explained by clinical and hemodynamic complications such as low cardiac output and tissue hypoxia associated with heart disease. Additionally, patients with heart disease might have been admitted to hospital in a more severe condition, unaware of any prior loss of function.^23^, ^24^

Our results indicate that the risk of new morbidity increases by 18% with each additional year of age. This heightened risk in older individuals is associated with the presence of more severe health problems. The reasons for hospitalization in these cases tend to be more serious and often have a poorer prognosis. Additionally, older children are more prone to accidents, which can result in more lasting injuries.

The present study found that the risk of new morbidity increased by 9% for every one‐unit increase in the PELOD2 score at admission. The pathophysiological processes potentially associated with clinical and laboratory markers of PELOD2, which are known to lead to functional loss, are low cardiac output, cardiorespiratory arrest, organic dysfunction inflammation and trauma.^10^ Meert et al. studied patients who presented these processes; 59.9% had new morbidity and 40.1% succumbed.^24^ In another study, the PELOD2 score was also associated with new morbidity, as short and long‐term FSS was used.^15^ Thus, PELOD2, as it involves variables associated with clinical severity, contributes to identifying patients at greater risk of functional loss and consequently, who would benefit when possible from support and rehabilitation not only during their stay in the ICU, but also during the period of clinical ward and after hospital discharge.^6^, ^12^, ^24^, ^25^

In alignment with another study, patients who developed new morbidity had the longest length of stay in the ICU.^6^ In fact, the longest length of stay in the ICU is associated with greater clinical complexity, which requires longer use of mechanical pulmonary ventilation (PMV), vasoactive drugs, sedation, analgesia, and renal and respiratory replacement therapies. These care situations hinder the use of rehabilitation therapies resulting in increased physical and cognitive problems, which in turn elevate the risk of new morbidity.^7^, ^12^, ^13^, ^14^, ^15^, ^16^, ^17^, ^18^, ^25^, ^26^, ^27^, ^28^

It is worth noting that this study had only one researcher who collected data of interest, who was previously trained to use the instruments accurately, including the FSS. Furthermore, to the best of our knowledge, this is the only multicenter study conducted in a medium‐income country that used FSS to quantify new morbidities. On the other hand, all variables potentially associated with this outcome were not used in the analyses, such as quality of life, anthropometric nutritional status, and available family social support network.^29^ Furthermore, the results of this study should be interpreted with caution by ICU practitioners from countries or regions with different socioeconomic characteristics.

Since new morbidity occurs in ICUs with relative frequency, and potentially leads to loss of quality of life in children and adolescents,^30^ it is recommended to adopt strategies to prevent functional loss, such as the flowchart of the “Surviving Sepsis Campaign 2020” new guidelines, with a focus on the management of sepsis and septic shock and their complications and, consequently, reduce the risk of dysfunction of multiple organs on a consistent and daily basis.^24^ A comprehensive view of the consequences for pediatric patients who survive ICU admission is fundamental within the concept of pediatric post‐intensive care syndrome, which requires appropriate referral and follow‐up after discharge to optimize the well‐being of both children and families.^31^

In this context, to reduce the risk of new functional morbidity during hospitalization, the care team should closely monitor the critically ill pediatric population, particularly those who are older or have chronic clinical conditions, especially heart diseases.^14^, ^22^, ^29^, ^30^, ^31^, ^32^

Finally, the availability of resources and use of care protocols can increase the efficiency of ICU care and should be considered by health managers. Likewise, regular and standardized assessments should be conducted during hospitalization to prevent new morbidity upon hospital discharge and in the long term.^32^

## AUTHOR CONTRIBUTIONS

Study design: TK, HPL, PKN, RMG; Data collection: RMG; Data analysis: TK, KCNA, PKN, and HPL; Manuscript writing: RMG, TK, and HPL; Manuscript revision: TK and HPL; Study supervision: TK, PKN, and HPL. All authors gave approval of the final version.

## CONFLICT OF INTEREST STATEMENT

The authors declare no conflict of interest.

## Data Availability

The database that originated the article is available upon request, with the corresponding author.
